# Crystallization of Amorphous Nimesulide: The Relationship between Crystal Growth Kinetics and Liquid Dynamics

**DOI:** 10.3390/molecules28072919

**Published:** 2023-03-24

**Authors:** Qin Shi, Yanan Wang, Jianfei Kong

**Affiliations:** 1 School of Pharmacy, Jiangsu Vocational College of Medicine, Yancheng 224005, China; 2 School of Pharmacy, Faculty of Health and Medical Science, Taylor’s University, Subang Jaya 47500, Selangor, Malaysia

**Keywords:** crystal growth, physical stability, molecular mobility, fragility, viscosity

## Abstract

Understanding crystallization and its correlations with liquid dynamics is relevant for developing robust amorphous pharmaceutical solids. Herein, nimesulide, a classical anti-inflammatory agent, was used as a model system for studying the correlations between crystallization kinetics and molecular dynamics. Kinetic parts of crystal growth (*u*_kin_) of nimesulide exhibited a power law dependence upon the liquid viscosity (*η*) as *u*_kin_~*η*^−0.61^. Bulk molecular diffusivities (*D*_Bulk_) of nimesulide were predicted by a force-level statistical–mechanical model from the α-relaxation times, which revealed the relationship as *u*_kin_~*D*_bulk_^0.65^. Bulk crystal growth kinetics of nimesulide in deeply supercooled liquid exhibited a fragility-dependent decoupling from *τ*_α_. The correlations between growth kinetics and α-relaxation times predicted by the Adam–Gibbs–Vogel equation in a glassy state were also explored, for both the freshly made and fully equilibrated glass. These findings are relevant for the in-depth understanding and prediction of the physical stability of amorphous pharmaceutical solids.

## 1. Introduction

Crystallization, a ubiquitous process, has attracted considerable interest in the field of pharmaceutical science. For the preparation of amorphous pharmaceutical solids, crystallization should be avoided during milling, solvent evaporation, melt-quenching, condensing of vapors, etc. Maintaining the drug in amorphous form during storage is also required to fully exert its advantages in solubility and dissolution rate, thus enhancing oral bioavailability. However, crystallization can be observed in amorphous pharmaceutical solids during preparation, storage, and dissolution, sometimes with extremely fast rates [1,2]. Moreover, our understanding of the mechanism of crystallization is still far from perfect.

Crystallization consists of two steps: nucleation and crystal growth. Compared to the nucleation process, crystal growth has been more intensively studied [2,3,4,5,6]. In the supercooled liquid, the crystal growth rates of a one-component amorphous system generally exhibit a bell-shaped curve as a result of competition between molecular mobility and the thermodynamic driving force. Bulk diffusion has been proposed to mainly control the crystal growth in the deep supercooling region, as evidenced by the proportional relationship between growth rates and bulk diffusion coefficients [7]. However, some studies have reported that the bulk diffusion-controlled growth model is invalid in some amorphous solids as temperature decreases near or below *T*_g_ [1,8]. One growth mode, termed the glass-to-crystal (GC) mode, has been reported in some organic molecules [1,9,10]. Upon decreasing the temperature near or below *T*_g_, a sudden increase in the crystal growth rate can be observed [8,9,10]. GC growth initially generally exhibits a fiber-like form as a precursor, and compact spherulites with further cooling [11,12]. In addition, GC growth has been reported to conserve the overall volume and form of the polycrystalline structure, showing the same unit cells as crystals grown in the supercooled liquid [10]. The activation energies of GC growth are similar to those of surface crystal growth [10]. The formation mechanisms of GC growth have been explained by several models, including surface-facilitated transformation, solid-state transformation by local mobility, and tension-induced interfacial molecular mobility. [10]. The other growth models, occurring at the free surface, have mainly been attributed to rapid surface molecular diffusion [2,13]. In a very recent study, Yu and co-workers found that this surface diffusion is controlled by bulk fragility, which is one measure widely used for evaluating how quickly dynamics are excited as a glass is heated to become a liquid [14].

In the present work, nimesulide was used as a model system to study the crystal growth kinetics and their correlations with molecular dynamics. The liquid dynamics of nimesulide were investigated by measuring the viscosity and relaxation time as a function of temperature. Bulk molecular diffusivities (*D*_Bulk_) were also predicted from the force-level statistical–mechanical model on the basis of the measured structural relaxation time (*τ*_α_). Correlations between crystal growth kinetics and liquid dynamics were evaluated to identify the key factor maintaining physical stability.

## 2. Experimental Section

### 2.1. Materials

Nimesulide was purchased from J&K Scientific Co. Ltd., Beijing China (purity > 99.0%) and used as received.

### 2.2. Measurement of the Crystal Growth of Nimesulide

The crystal growth kinetics of nimesulide were calculated by measuring the growth front as a function of time under a polarized light microscope (XP-L2000A, Shanghai Millimeter Precision Instrument Co., Ltd., Shanghai, China). A hot stage (KELX-4A, Shanghai Millimeter Precision Instrument Co., Ltd., Shanghai, China) was used to precisely control the temperature for crystal growth. A total of 3–5 mg crystalline nimesulide was melted between two 15 mm-diameter round coverslips at 155 °C for 5 min. Subsequently, amorphous nimesulide was obtained by quenching the samples to room temperature by contact with an aluminum block. For the crystal growth at the free surface, the surface of the amorphous nimesulide solid was exposed by detaching one coverslip from the samples. To measure the crystal growth of the nimesulide above 40 °C, the melt-quenched samples were placed on the hot stage at the desired experimental temperature. For comparisons, growth rates of nimesulide below 40 °C were measured by storing these samples inside the desiccators placed in an oven or refrigerator and precisely controlling the experimental temperature. For measurement of the steady-state growth rate, we ensured that the plot of length change vs. time was linear. Each reported crystal growth rate was obtained by calculating the average of four measurements.

### 2.3. Raman Microscopy

A ThermoFisher DXR Raman microscope (Madison, WI, USA) was used to identify the polymorph of crystalline nimesulide grown in the interior and at the free surface. The Raman spectra were obtained over a 1800–1850 cm^−1^ wavelength range and 16 mW laser power, using a 1 s exposure time, repeated 15 times. Nimesulide has been reported to have two polymorphs, with the melting points at 147 and 143 °C, respectively. Herein, nimesulide crystals observed in the interior or at the free surface were all identified by Raman spectra as form I, the thermodynamically stable polymorph. 

### 2.4. Thermal Analysis

A TA Q2000 differential scanning calorimeter (DSC) (New Castle, DE, USA) under a 50 mL/min nitrogen purge was used for thermal analysis of nimesulide. A total of 3–5 mg of the crystalline form I of nimesulide was loaded in an aluminum pan. These samples were first heated up to 160 °C (13 °C higher than the *T*_m_ of nimesulide form I, with a melting point at 147 °C) at a heating rate of 10 °C/min and annealing for 3 min for complete melting of the crystalline form. Then, the samples were cooled to 0 °C, with a cooling rate of 20 °C/min. Subsequently, the samples were reheated to 160 °C at a rate of 10 °C/min.

### 2.5. Broadband Dielectric Spectroscopy

The molecular mobility of amorphous nimesulide was measured by a Concept 80 Novocontrol Alpha broadband dielectric spectrometer (GmbH & Co. KG, Germany) the frequency range of 10^−1^–10^6^ Hz. Melt-quenched nimesulide was placed tightly between two copper-coated brass electrodes (20 mm diameter). A 0.5 mm-thick PTFE circular ring spacer was used to avoid the overflow of nimesulide liquid. We measured the complex dielectric permittivity ε*(*ω*), which consisted of real part *ε*′ and imaginary part *ε*″, as a function of angular frequency *w* and temperature *T*. To determine the average relaxation time (τ_HN_), the obtained spectra were analyzed using the Havriliak−Negami (HN) function plus dc-conductivity term [15,16]:

(1) $$\varepsilon^{*}\left(\omega \right)=\varepsilon^{\prime }\left(\omega \right)-i\varepsilon^{\prime \prime }\left(\omega \right)=\varepsilon_{\infty }+\frac{\varepsilon_{s}-\varepsilon_{\infty }}{{\left(1+{\left(i\omega \tau_{HN}\right)}^{\alpha }\right)}^{\beta }}+\frac{\sigma_{dc}}{i\omega \varepsilon_{0}}$$

where *ε*_∞_ represents high-frequency limit permittivity and *ε*_s_ is the static dielectric constant. *α* and *β* are shape parameters of dielectric peaks, which, respectively, represent the asymmetry and width. *σ*_dc_/*iε*_0_ω represents the conductivity component, where *σ*_dc_ represents the level of dc-conductivity and *ε*_0_ is the permittivity of the vacuum.

The α-relaxation time (τ_α_) was calculated by the following equation, on the basis of the parameters obtained by the HN function [15,16].

(2) $$\tau_{\alpha }=\tau_{\max}=\tau_{\mathrm{HN}}{\left[\sin\left(\frac{\pi \alpha }{2+2\beta }\right)\right]}^{-1/\alpha }{\left[\sin\left(\frac{\pi \alpha \beta }{2+2\beta }\right)\right]}^{1/\alpha }$$

An empirical Vogel–Flucher–Tammann (VFT) equation (Equation (3)) is most frequently used for describing the temperature dependencies of the α-relaxation time [17]:

(3) $$\tau =\tau_{0}\exp\left(\frac{DT_{0}}{\left(T-T_{0}\right)}\right)$$

where *τ* is the average α-relaxation time, and *τ*_0_ is the relaxation time of the unrestricted material (10^−14^ s, the quasi lattice vibration period). *T*_0_ represents the zero-mobility temperature and *D* is one of the strength parameters, specifically a measure of fragility.

### 2.6. Shear Viscosity Measurement

The steady shear viscosities of amorphous nimesulide were measured using an ARES G2 rheometer (TA Instruments). The viscosity measurements were conducted in two parallel plates (diameter: 25 mm) in oscillatory mode, and the gap between the two parallel plates was 0.4 mm. The crystalline nimesulide powders were first melted on the lower parallel plate at 160 °C for 5–10 min to ensure complete melting. The upper plate was then lowered, and the temperature was lowered to the desired temperatures for measurements. A shear deformation was applied at a rate of 1 s^−1^ and the values of shear viscosity were obtained when the rate reading became steady.

## 3. Results and Discussion

### 3.1. Correlations of Crystal Growth Kinetics and the Molecular Mobility of Nimesulide in the Supercooled Liquid

**Figure 1:**
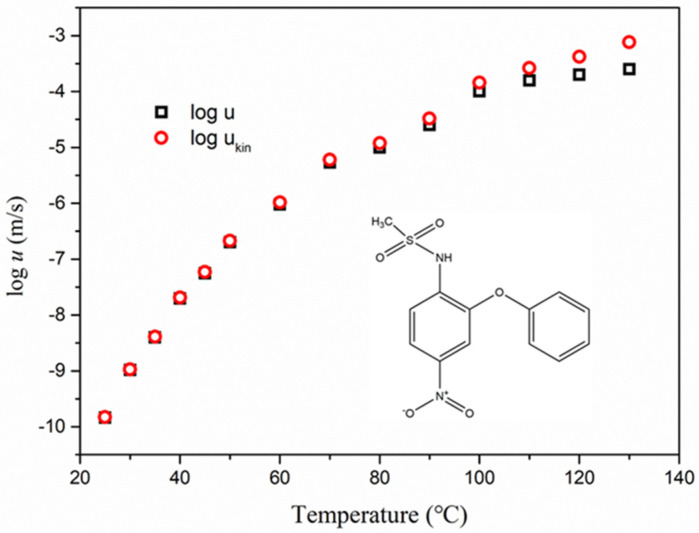
Crystal growth rates of nimesulide in supercooled liquid (black empty square). This plot was reconstructed from [8]. The empty red circles represent the kinetic part of the growth rate *u*_kin_ as a function of temperature. The inset is the molecular structure of nimesulide.

Figure 1 shows the measured crystal growth rate *u* of nimesulide as a function of temperature and the calculated kinetic part of the growth rate *u*_kin_. Herein, *u*_kin_ is defined in terms of growth rate *u* [18].

(4) $$u_{kin}=u/\lfloor 1-\exp\left(-△S_{m}△T/RT\right)\rfloor$$

△*S*_m_ is the melt-crystalline entropy difference evaluated at *T*_m_ and △*T* represents the undercooling. The value of △*S*_m_ measured in our study was very close to that reported in a previous study [19]. As observed in Figure 1, the crystal growth rates of nimesulide below the maximum in *u* were dominated by *u*_kin_, particularly in the deep supercooled region. *u*_kin_ is relevant to the activated process of molecules in supercooled liquid to organize the crystal phase [18,20].

**Figure 2:**
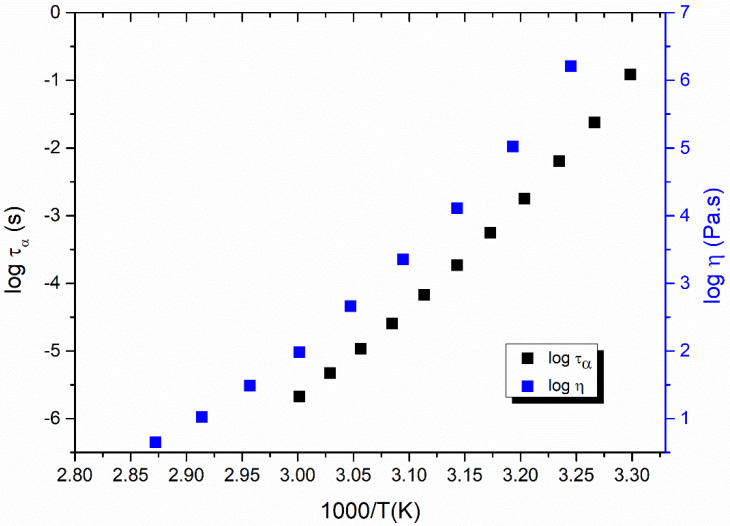
Temperature dependence of the α-relaxation time and viscosity of pure nimesulide. The black squares represent the α-relaxation time of nimesulide and were reconstructed from our previous study [24]. The blue squares represent the viscosity of nimesulide measured in the present study.

It is widely accepted that molecular mobility is probably the most relevant factor for controlling the crystal growth of amorphous solids [17,21,22]. In present study, in order to reveal the relationships between liquid dynamics and the kinetic part of the growth rate, the viscosity of nimesulide was carefully measured with a rheometer. Figure 2 shows the shear viscosity of nimesulide as a function of temperature. The viscosity of nimesulide is determined when the supercooled liquid rises quickly with decreasing temperature towards the glass transition temperature. A magnitude of increase of nearly five orders can be observed in the viscosity of nimesulide with a decrease in temperature from 80 °C to 35 °C.

Broadband dielectric spectroscopy has been also widely used to examine the relaxation processes originating from various kinds of molecular mobility in recent decades [23]. In our previous study, the molecular mobility of nimesulide with or without the addition of profen analogs was investigated by broadband dielectric spectroscopy [24]. The addition of profen analogs strongly affected the crystallization kinetics and molecular dynamics of amorphous nimesulide. However, the relationship between crystallization kinetics and liquid dynamics has not received in-depth investigation for the pure amorphous drug system. Knapik et al. investigated the molecular dynamics and physical stability of amorphous nimesulide in or without the presence of three well-known polymers: inulin, Soluplus, and PVP. Isothermal and non-isothermal kinetics and activation energies of crystallization of these polymer-doped systems were investigated and measured. PVP, the best stabilizer among these three polymers, was reported to prolong the crystallization half-life time of pure nimesulide at 55 °C from ~33 min to ~40 years by adding 20% *w*/*w* PVP. These inhibitory effects on crystallization of nimesulide can be mainly attributed to polymeric steric hindrances and the anti-plasticization effect exerted by the addition of the polymer. In the present study, focused on the relationship between crystal growth kinetics and liquid dynamics from both the perspective of relaxation time and viscosity.

Analogous to the α-relaxation time, the viscosity of nimesulide also deviates from the Arrhenius temperature dependence. As shown in Figure 2, the shear viscosity of nimesulide supercooled liquid generally couples with the α-relaxation time. α-relaxation time is a measure of molecular mobility, which is defined as the time required for the reorientation of entire molecules for low-molecular-weight materials. The α-relaxation times obtained by the dielectric technique only represent the rotational motions, which indicates the coupling between the rotational motions and shear viscosity of nimesulide. In addition, the shear viscosity of nimesulide also shows a slight decoupling from the α-relaxation time. This deviation may be attributed to the diversity of viscosity measurements, which might produce a small deviation when relating oscillatory flow to the steady shear flow in viscosity measurement.

**Figure 3:**
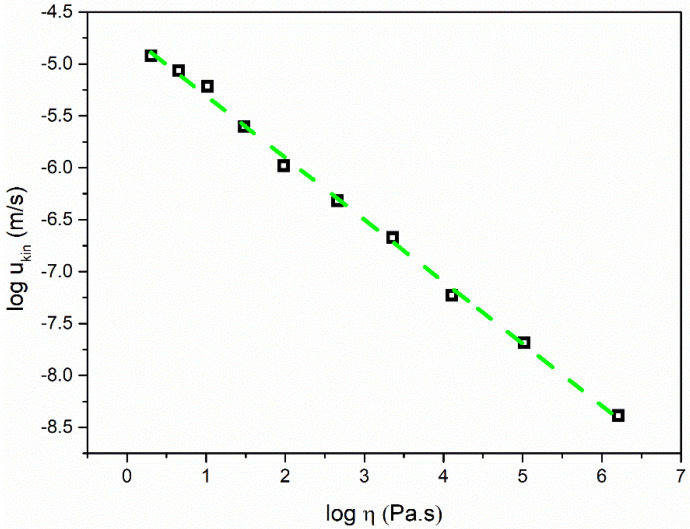
Kinetic crystal growth rate *u*_kin_ plotted against the viscosity of nimesulide. *u*_kin_ was obtained from Equation (4) and reconstructed from Figure 1.

Figure 3 shows the *u*_kin_ as a function of the liquid viscosity in a log–log format, where supercooled liquid nimesulide is approximately described by a straight line in the intermediate and deep supercooled region. Therefore, *u*_kin_ can be described as exhibiting a power law dependence upon the liquid viscosity:

(5) $$u_{kin}\;\propto \;\eta^{-\xi_{a}}$$

where *ξ* is the decoupling exponent, which has been demonstrated to strongly relate to the liquid fragility in a single component liquid. The value of the exponent ξ_a_ was 0.61 for liquid nimesulide. Weaker temperature dependences of the crystal growth rates of nimesulide can also be observed in comparison with the viscosity. These results are strongly related to the spatially heterogeneous dynamics near *T*_g_ [7]. A significant enhancement can be expected in the translational self-diffusion in comparison to the viscosity near *T*_g_, which will far exceed the expectations of the Stokes–Einstein equation [7].

**Figure 4:**
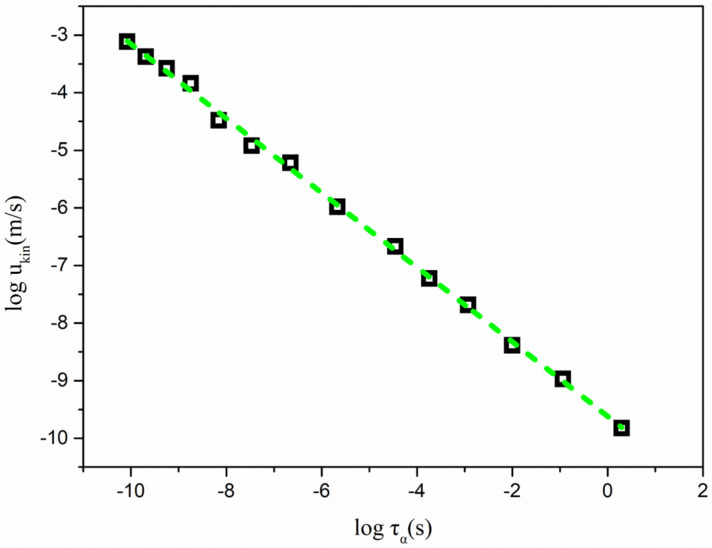
*u*_kin_ plotted against *τ*_α_ of pure nimesulide as a function of temperature. *u*_kin_ was obtained from Equation (4) and reconstructed from Figure 1. *τ*_α_ was calculated from the VFT equation extending to the higher temperature of 140 °C.

Figure 4 shows *u*_kin_ as a function of *τ*_α_ in the log–log format. A linear correlation was observed between the *u*_kin_ and *τ*_α_ of pure nimesulide in the temperature range of 25–140 °C in a log–log format. Investigating the linear correlations facilitated the exploration of the relationship between crystal growth and molecular dynamics. An equation was used to represent the linear correlations, as follows [20]:

(6) $$log_{10}u_{kin}=-\xi_{b}log_{10}\tau_{\alpha }+A$$

where *ξ*_b_ is a measure of the coupling relationship for the kinetic part of the growth rates and α-relaxation times. If the value of *ξ*_b_ is close to 1, it is suggested that the kinetic parts of crystal growth can mainly be attributed to the global molecular mobility represented by the α-relaxation times. For the comparisons, a smaller value of *ξ*_b_ indicated that the global mobility was less responsible for the kinetic part of the crystal growth of nimesulide. The correlations of the abovementioned crystallization process and molecular mobility can also be described as a power law of *u*_kin_ upon α-relaxation times.

(7) $$u_{kin}\;\propto \;\tau_{\alpha }^{-\xi_{b}}$$

The value of *ξ*_x_ was calculated as ~0.65. The value of *ξ*_b_ was close to that reported in the griseofulvin system, but smaller than those of indomethacin polymorphs [18,20]. Ediger and co-workers reported that the values of *ξ* are strongly correlated with the fragility of the liquid [18]. Fragility, proposed by Martinez and Angell, quantifies the extent of viscosity away from those systems exhibiting an Arrhenius temperature dependence. Fragility is also one of the important measures of the ease with which dynamics are excited upon heating a glass to form a liquid. A strong system effectively resists excitation, while a fragile system gains mobility when the glass is heated above *T*_g_. In the case of SiO_2_, its strong character originates from the robustness of the three-dimensional network, consisting of covalent bonds. The fragile character of some systems result from the rapid unraveling of the local molecular structure.

Böhmer et al. defined the fragility of glass formers as follows [25]:

(8) $$m=\frac{dlog\tau_{\alpha }}{d(\frac{T_{g}}{T})}\;┃T=T_{g}=\frac{D(\frac{T_{0}}{T_{g}})}{(1-{\left(T_{0}/T_{g}\right)}^{2}ln\left(10\right)}$$

The parameters in Equation (8) can be obtained from the empirical VFT equation. The fragility parameter m of pure nimesulide liquid was calculated as ~84.6, which can be classified as the intermediate glass former. This value for the fragility parameter of nimesulide is similar to that reported by Knapik et al. [26]. In general, the fragility parameter can be used to predict the glass-forming ability and recrystallization tendency of amorphous solids [26,27,28]. On the basis of the two-order parameter model, it is expected that strong materials with a higher fragility parameter are more physically stable than fragile materials [29]. This can mainly be attributed to the frustration of strong materials against re-crystallization. Frustration of amorphous solids originates from the competition between long-range ordering and short-range ordering. The former is responsible for nucleation and crystal growth processes. Short-range ordering is relevant for the formation of local favored structures, which exhibit no crystallographic symmetry.

**Figure 5:**
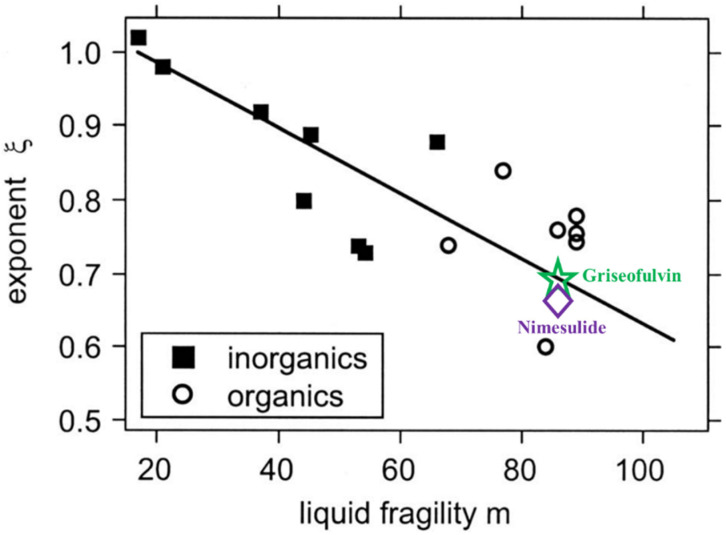
Exponent *ξ*_x_ is plotted as a function of fragility of organic and inorganic supercooled liquids. The plot was reconstructed from [18]. The green hollow pentagram represents the griseofulvin data in our previous study. The purple diamond represents the nimesulide in the present work. Adapted from the work of Ediger et al. (2008) with permission. Copyright 2008 American Institute of Physics.

Ediger et al. proposed that crystal growth kinetics decouple from viscosity in a manner that strongly depends on the fragility of the supercooled liquid [18]. A linear correlation between *ξ*_x_ and fragility *m* can be approximately described as *ξ* ≈ 1.1–0.005 *m*. Shi and co-workers tested the generality of the abovementioned relationships between the decouple exponent and fragility by using griseofulvin as a model system [20]. The values of the decouple exponent and fragility were obtained by analyzing the kinetic part of the crystal growth rate and α-relaxation time, rather than the liquid viscosity [20]. As shown in Figure 5, the relationship between the decouple exponent and fragility of griseofulvin corroborated the findings of Ediger et al. It is accepted that fragility is explicitly a property largely determined by liquid dynamics, irrespective of the crystal state. This finding further supports the view that the kinetic part of crystal growth in intermediate and deep supercooled regions is mainly controlled by liquid dynamics. The exponent *ξ*_x_ and liquid fragility *m* of nimesulide also fit the linear correlation proposed by Ediger et al. Our findings also supported the view that crystal growth could be a new window for investigating the liquid dynamics in supercooled liquids.

Mirigian and Schweizer defined one hopping diffusivity *D*_bulk_ for bulk homogeneous material as follows [30]:

(9) $$D_{bulk}=\frac{d^{2}}{6\tau_{\alpha }}$$

**Figure 6:**
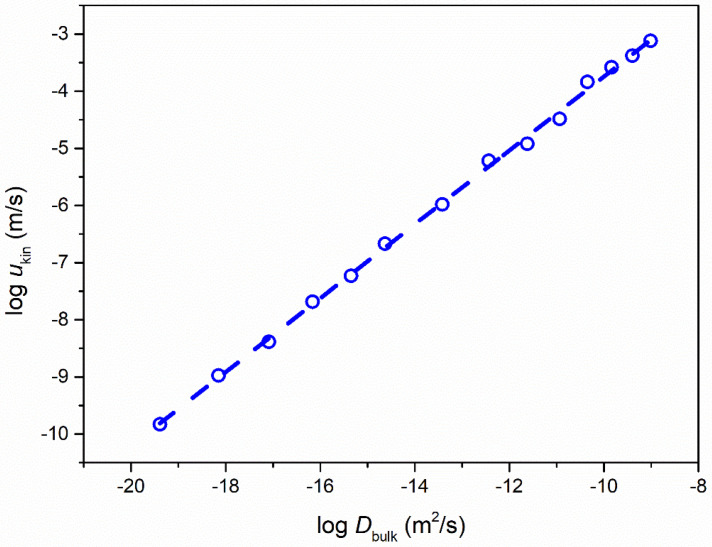
*u*_kin_ plotted against *D*_bulk_ of pure nimesulide as a function of temperature. *D*_bulk_ was calculated from α-relaxation time based on Equation (8).

*d* can approximately represent the molecular diameter. Sun et al. proposed that the molecular diameter can be approximately calculated by treating the molecules as spheres packed closely together to occupy 74% of the space [11]. On the basis of density and molecular weight, the molecular diameter d can be calculated as

(10) $$d=1.12\;V^{1/3}$$

where V represents the unit cell volume per molecule. The molecular diameter of nimesulide was calculated as 7.0 Å. Figure 6 shows *u*_kin_ as a function of *D*_bulk_ in the log–log format. The overall trend (dotted line) was accurately described by the power law as *u*_kin_~*D*_bulk_^0.65^.

As reported in our previous study, rapid crystal growth on the free surface could be clearly observed in the supercooled liquid of nimesulide [8]. Unlike that reported in griseofulvin, the surface crystal growth rate of nimesulide exhibited a slight jump at 30–40 °C. Similar results were also observed for the surface crystal growth of felodipine. The surface crystal growth of nimesulide was separated into two continuous parts (i.e., T ≤ 30 °C and T ≥ 35 °C). It is widely accepted that liquid flow above *T*_g_ disrupts the surface crystallization in supercooled liquids, and this effect is more serious with increasing temperature. Therefore, single liquid dynamic cannot fully explain or predict surface crystallization of nimesulide at higher temperatures in the supercooled liquid.

### 3.2. Correlations of Crystal Growth Kinetics and Molecular Mobility of Nimesulide in the Glassy State

It is widely accepted that the α-relaxation time cannot be fully responsible for crystal growth in the glassy state, particularly for a system exhibiting rapid glass-to-crystal (GC) growth or surface crystal growth. GC growth, a special mode activated near *T*_g_, leads to orders of magnitude faster growth rates than those predicted by a bulk diffusion-controlled growth model. Sun et al. investigated the relationship between crystal growth of 5-methyl-2-[(2-nitrophenyl)amino]-3-thiophenecarbonitrile (ROY) polymorphs and α-relaxation times [31]. In the case of the GC growth behavior of ROY polymorphs, the growth rates are too fast to be controlled by the a-relaxation process [32]. Similar results have also been observed in the GC growth of griseofulvin [20]. Nimesulide also exhibits rapid GC growth in the glassy state. As the temperature decreases from 25 °C (*T*_g_ + 2 °C) to 18 °C (*T*_g_ − 5 °C), an increase greater than one order of magnitude can be observed in the crystal growth rates of nimesulide.

In recent decades, secondary relaxation has also attracted considerable attention in exploration of the underlying mechanism of crystallization in the glassy state [20,32]. In the case of amorphous nimesulide, α-relaxation is too slow to be observed in the dielectric spectra collected below *T*_g_ [26]. Only one secondary relaxation can be observed [26]. Analogous to the α-relaxation mode, this secondary relaxation moves towards higher frequencies with an increase in temperature. However, β-relaxation times are found to exhibit less temperature dependence in comparison with α-relaxation times. Secondary relaxations mainly result from local molecular motions. Among the secondary relaxations, the Johari–Goldstein (JG) process, representing fast local reorientations of entire molecules, has attracted considerable interest for its role in physical stability in the glassy state. Moreover, it has also been proposed that JG relaxation is the precursor to glass transition and molecular mobility of cooperative α-relaxation.

For nimesulide, the temperature dependence of β-relaxation has been reported to be fitted to the Arrhenius equation [17]:

(11) $$\tau_{\beta }=\tau_{\infty }\exp(\frac{△E_{\beta }}{k_{B}T})$$

where τ_∞_ represents the pre-exponential factor and is calculated as 6.31 × 10^−15^ s. △E*_β_* represents the energy barrier for the examined β relaxation process, whose calculated value is 41.9 KJ/mol for a pure nimesulide amorphous system. *k*_B_ is the Boltzmann constant.

**Figure 7:**
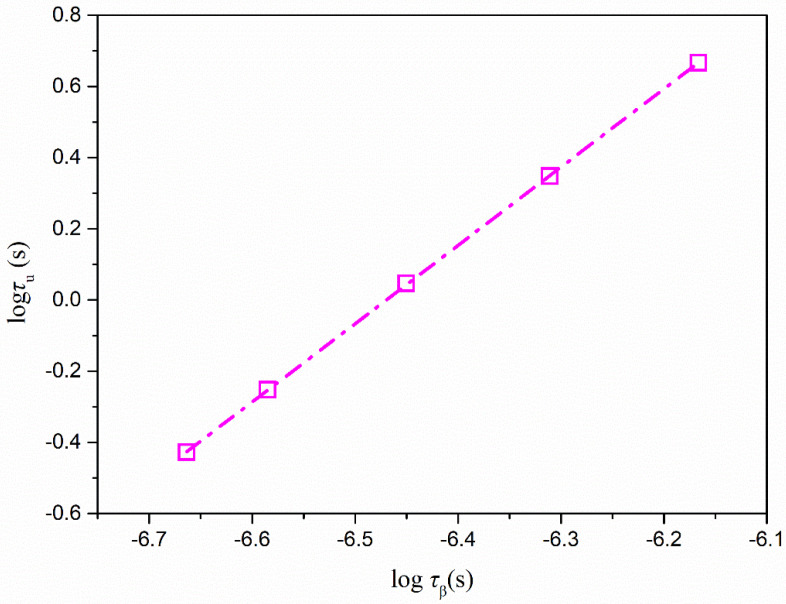
τ_u_ plotted against *τ*_β_ of pure nimesulide as a function of temperature in the glassy state (0, 5, 10, 15, 18 °C). The secondary relaxation time of pure nimesulide was calculated from Equation (10) and the value obtained from [26].

In order to reveal the potential relationship between crystallization in the glassy state and secondary relaxation times, a linear correlation was established by the following equation [17]:

(12) $$\log\tau_{u}=\xi_{X}\log\tau_{\beta }+A$$

where τ_u_ represents the time for a nimesulide crystal to grow one layer of molecules. *τ*_u_ = *a*/*u*, where *a* is the calculated diameter of nimesulide as ~7.0 Å, and *u* represents the growth rates of nimesulide measured in the glassy state. The coupling coefficient *ξ*_x_ is a measure of the coupling relationship of *τ*_u_ and *τ*_β_. If the value of *ξ*_x_ is close to 1, it is proposed that the abovementioned crystallization processes can mainly be attributed to *τ*_β_. A smaller value of *ξ*_x_ indicates that *τ*_β_ is less responsible for these crystallization processes. Coupling coefficient *ξ*_x_ reflects the ratio of the activation energies of the abovementioned crystallization and molecular relaxation *τ*_β_. Molecular relaxations should not trigger the crystallization process if the ratio is above 1. Figure 7 shows the *τ*_u_ as a function of *τ*_β_ in a log–log format. In the case of nimesulide, *ξ*_x_ was calculated as 2.20, indicating no links between τ_u_ and *τ*_β_. Some researchers have proposed that secondary relaxation could provide the molecular motions responsible for glass-to-crystal growth in the glassy state [12]. A shorter characteristic time of secondary relaxation can be observed in comparison with that of GC growth. In addition, a similar activation energy of characteristic time of secondary relaxation as GC growth has also been reported in some organic systems. However, in the present study, no direct evidence was found supporting the view that GC growth is controlled by secondary relaxation.

Some studies have also proposed that secondary relaxation processes are apparently irrelevant to GC growth. In the case of glassy o-terphenyl, the secondary relaxation peak measured by dielectric spectroscopy disappeared upon aging. However, GC growth could still be clearly observed in glassy o-terphenyl. Sun et al. investigated the growth behavior of ROY polymorphs, as well as the liquid dynamics [32]. GC growth was found in some polymorphs of ROY, while no secondary relaxation peaks could be observed [32]. They proposed that the observation of secondary relaxation might not be a necessary condition for the existence of GC growth.

In the present work, we also considered the α-relaxation time as a potential contributor to controlling the physical stability of amorphous solids in the glassy state. However, α-relaxation times are challenging to experimentally measure by dielectric spectroscopy due to their extremely long time scale. One Adam–Gibbs–Vogel (AGV) equation is commonly used to predicting the α-relaxation time in the glassy state [17,33]:

(13) $$\tau =\tau_{0}\exp(\frac{DT_{0}}{\left(T(1-\frac{T_{0}}{T_{f}}\right)})$$

where *τ*_0_, *D*, and *T*_0_ can be obtained from the VFT equation. *T*_f_ is the fictive temperature, which is defined as the temperature where the value of configuration entropy is the equilibrium value. For the freshly prepared glass, *T*_f_ can often be estimated to be equal to *T*_g_ for the sake of simplicity [17,34]. It is widely accepted that the glass state is nonequilibrium and keeps aging towards the equilibrium state. A dramatic increase in α-relaxation time is expected during the continuous non-linear and non-exponential aging process. For fully equilibrated glass (aging time = ∞), *T*_f_ is applied as *T*, and the AGV equation reduces to the VFT equation [17,34].

**Figure 8:**
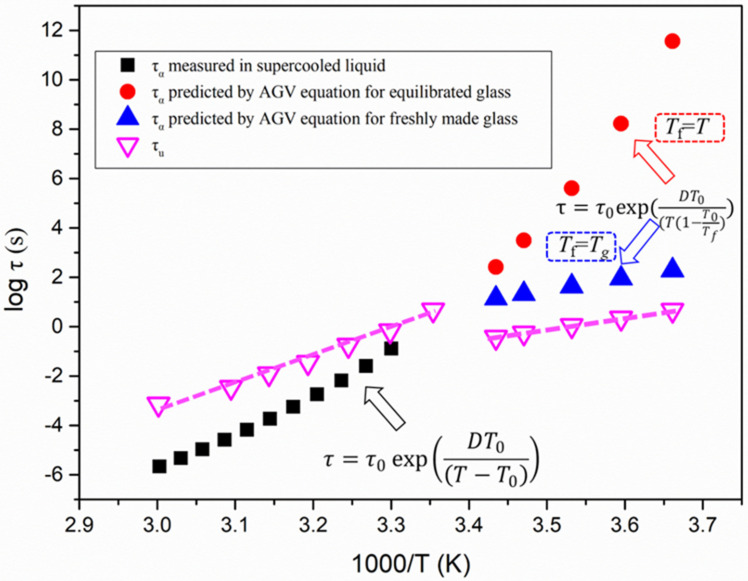
Temperature dependence of α-relaxation times for pure nimesulide and *τ*_u_. The black squares represent the α-relaxation times obtained by the VFT equation (Equation (4)). The red rounds and blue triangles represent the α-relaxation times obtained from the AGV equation (Equation (12)) for the fully equilibrated glass and freshly made glass. The purple empty inverted triangles represent τ_u_ for pure nimesulide in the glassy and supercooled liquid region.

Figure 8 compares the τ_α_ of liquid and glass of nimesulide with *τ*_u_, the characteristic time for its crystal growth. For the crystal growth of nimesulide in the supercooled liquid region, the growth rate was generally characterized as τ_u_ > τ_α_. With decreasing temperature it entered the glassy state, and the τ_u_ of nimesulide was much shorter in comparison with τ_α_ for both freshly made and fully equilibrated glass. These results suggest that GC growth of nimesulide in the glassy state is too rapid to be under the control of α-relaxation. As shown in Figure 8, at the time scale of one *τ*_α_ in the glassy state, more than 30 molecular layers of nimesulide are added into the crystalline phase. Analogous results have been found in griseofulvin and ROY polymorphs showing GC growth [20,31]. In the case of griseofulvin, it has been reported that hundreds of griseofulvin molecular layers would be added to its crystalline phase [20]. We also explored the possible relationship between surface crystal growth and the α-relaxation process of pure nimesulide. In our previous study, crystals of nimesulide grew much more rapidly on the free surface in comparison with the interior [8]. In addition, surface crystal growth of nimesulide in the glassy state exhibited approximately similar temperature dependence [8]. It is expected that the *τ*_u_ of the surface crystal growth of nimesulide would be much smaller than *τ*_α_.

Surface crystal growth, which can be halted by a thin surface coating of a few nm, is proposed to be mainly attributed to high surface mobility [2,35]. The establishment of a link between surface mobility and crystallization is highly important because crystallization can cause the failure of amorphous formulations. The velocity of surface crystal growth was also demonstrated to be approximately proportional to the coefficient of surface diffusion *D*_s_ [13]. With the increase in the molecular size and strength of hydrogen bonding interactions, the surface diffusion is slowed down [36,37]. In a very recent study, Yu and co-workers proposed that surface diffusion is controlled by bulk fragility [14]. This correlation originated from the robustness of covalent network bonds for strong liquids, which make them more resistant to environmental excitation from the bulk to free surface. This is helpful for understanding and predicting the surface mobility, in order to develop robust amorphous formulations. Further work should also pay more attention to predicting surface mobility based on the liquid dynamics in the interior of amorphous formulations.

## 4. Conclusions

This work systemically investigated the correlation between crystallization and liquid dynamics of nimesulide in the supercooled liquid and glassy state. The exponent *ξ*_x_ and liquid fragility *m* of nimesulide fit the linear correlation proposed by Ediger et al., which indicated that the kinetic part of the bulk crystal growth of nimesulide *u*_kin_ in the supercooled liquid exhibited fragility-dependent decoupling from *τ*_α_ and viscosity. In addition, the relationship between *u*_kin_ and the predicted *D*_bulk_ was described by the power law as *u*_kin_~*D*_bulk_^0.65^. In the glassy state, no link was observed between the secondary relaxation time and crystallization in the interior. Moreover, GC growth and rapid surface growth were demonstrated to be too fast to be under the control of the α-relaxation process for either freshly made or fully equilibrated glass.

## Data Availability

Not applicable.

## References

[1] Shi Q., Li F., Yeh S., Wang Y., Xin J. (2020). Physical stability of amorphous pharmaceutical solids: Nucleation, crystal growth, phase separation and effects of the polymers. Int. J. Pharm..

[2] Yu L. (2016). Surface mobility of molecular glasses and its importance in physical stability. Adv. Drug Deliv. Rev..

[3] Sun Y., Zhu L., Wu T., Cai T., Gunn E.M., Yu L. (2012). Stability of amorphous pharmaceutical solids: Crystal growth mechanisms and effect of polymer additives. AAPS J..

[4] Huang C., Powell C.T., Sun Y., Cai T., Yu L. (2017). Effect of low-concentration polymers on crystal growth in molecular glasses: A controlling role for polymer segmental mobility relative to host dynamics. J. Phys. Chem. B.

[5] Wang K., Sun C.C. (2019). Crystal growth of celecoxib from amorphous state: Polymorphism, growth mechanism, and kinetics. Cryst. Growth Des..

[6] Shi Q., Tao J., Zhang J., Su Y., Cai T. (2019). Crack- and bubble-induced fast crystal growth of amorphous griseofulvin. Cryst. Growth Des..

[7] Swallen S.F., Ediger M.D. (2011). Self-diffusion of the amorphous pharmaceutical indomethacin near Tg. Soft Matter.

[8] Shi Q., Wang Y., Xu J., Liu Z., Chin C. (2022). Fast crystal growth of amorphous nimesulide: Implication of surface effects. Acta Crystallogr. Sect. B Struct. Sci. Cryst. Eng. Mater..

[9] Shi Q., Moinuddin S.M., Wang Y., Ahsan F., Li F. (2022). Physical stability and dissolution behaviors of amorphous pharmaceutical solids: Role of surface and interface effects. Int. J. Pharm..

[10] Powell C.T., Xi H., Sun Y., Gunn E., Chen Y., Ediger M.D., Yu L. (2015). Fast crystal growth in o-terphenyl glasses: A possible role for fracture and surface mobility. J. Phys. Chem. B.

[11] Sun Y., Xi H., Ediger M.D., Yu L. (2008). Diffusionless crystal growth from glass has precursor in equilibrium liquid. J. Phys. Chem. B.

[12] Sun Y., Xi H., Chen S., Ediger M.D., Yu L. (2008). Crystallization near glass transition, transition from diffusion-controlled to diffusionless crystal growth studied with seven polymorphs. J. Phys. Chem. B.

[13] Huang C., Ruan S., Cai T., Yu L. (2017). Fast surface diffusion and crystallization of amorphous griseofulvin. J. Phys. Chem. B.

[14] Li Y., Annamareddy A., Morgan D., Yu Z., Wang B., Cao C., Perepezko J.H., Ediger M.D., Voyles P.M., Yu L. (2022). Surface diffusion is controlled by bulk fragility across all glass types. Phys. Rev. Lett..

[15] Havriliak S., Negami S. (1967). A complex plane representation of dielectric and mechanical relaxation processes in some polymers. Polymer.

[16] Iglesias T.P., Carballo M., Fernandez J.P. (2003). Broadband Dielectric Spectroscopy.

[17] Grzybowska K., Capaccioli S., Paluch M. (2016). Recent developments in the experimental investigations of relaxations in pharmaceuticals by dielectric techniques at ambient and elevated pressure. Adv. Drug Deliv. Rev..

[18] Li F., Xin J., Shi Q. (2021). Diffusion-controlled and ‘diffusionless’ crystal growth: Relation between liquid dynamics and growth kinetics of griseofulvin. J. Appl. Crystallogr..

[19] Ediger M.D., Harrowell P., Yu L. (2008). Crystal growth kinetics exhibit a fragility-dependent decoupling from viscosity. J. Chem. Phys..

[20] Baird J.A., Van Eerdenbrugh B., Taylor L.S. (2010). A classification system to assess the crystallization tendency of organic molecules from undercooled melts. J. Pharm. Sci..

[21] Knapik-Kowalczuk J., Rams-Baron M., Paluch M. (2021). Current research trends in dielectric relaxation studies of amorphous pharmaceuticals: Physical stability, tautomerism, and the role of hydrogen bonding. TrAC-Trend Anal. Chem..

[22] Svoboda R., Košťálová D., Krbal M., Komersová A. (2022). Indomethacin: The interplay between structural relaxation, viscous flow and crystal growth. Molecules.

[23] Wang Y., Wang Y., Cheng J., Chen H., Xu J., Liu Z., Shi Q., Zhang C. (2021). Recent advances in the application of characterization techniques for studying physical stability of amorphous pharmaceutical solids. Crystals.

[24] Zhang J., Shi Q., Qu T., Zhou D., Cai T. (2021). Crystallization kinetics and molecular dynamics of binary coamorphous systems of nimesulide and profen analogs. Int. J. Pharm..

[25] Böhmer R., Ngai K.L., Angell C.A., Plazek D.J. (1993). Nonexponential relaxations in strong and fragile glass formers. J. Chem. Phys..

[26] Knapik J., Wojnarowska Z., Grzybowska K., Tajber L., Mesallati H., Paluch K.J., Paluch M. (2016). Molecular dynamics and physical stability of amorphous nimesulide drug and its binary drug-polymer systems. Mol. Pharm..

[27] Kawakami K., Harada T., Yoshihashi Y., Yonemochi E., Terada K., Moriyama H. (2015). Correlation between glass-forming ability and fragility of pharmaceutical compounds. J. Phys. Chem. B.

[28] Knapik J., Wojnarowska Z., Grzybowska K., Jurkiewicz K., Tajber L., Paluch M. (2015). Molecular dynamics and physical stability of coamorphous ezetimib and indapamide mixtures. Mol. Pharm..

[29] Tanaka H. (2005). Relationship among glass-forming ability, fragility, and short-range bond ordering of liquids. J.Non-Cryst. Solids.

[30] Mirigian S., Schweizer K.S. (2015). Theory of activated glassy relaxation, mobility gradients, surface diffusion, and vitrification in free standing thin films. J. Chem. Phys..

[31] Bhattacharya S., Suryanarayanan R. (2009). Local mobility in amorphous pharmaceuticals—Haracterization and implications on stability. J. Pharm. Sci..

[32] Sun Y.X., Xi H., Ediger M.D., Richert R., Yu L. (2009). Diffusion-controlled and diffusionless crystal growth near the glass transition temperature: Relation between liquid dynamics and growth kinetics of seven ROY polymorphs. J. Chem. Phys..

[33] Shi Q., Zhang C., Su Y., Zhang J., Zhou D., Cai T. (2017). Acceleration of crystal growth of amorphous griseofulvin by low-concentration poly(ethylene oxide): Aspects of crystallization kinetics and molecular mobility. Mol. Pharm..

[34] Kothari K., Ragoonanan V., Suryanarayanan R. (2014). Influence of molecular mobility on the physical stability of amorphous pharmaceuticals in the supercooled and glassy States. Mol. Pharm..

[35] Priemel P.A., Laitinen R., Barthold S., Grohganz H., Lehto V.P., Rades T., Strachan C.J. (2013). Inhibition of surface crystallisation of amorphous indomethacin particles in physical drug-polymer mixtures. Int. J. Pharm..

[36] Zhang W., Yu L. (2016). Surface diffusion of polymer glasses. Macromolecules.

[37] Chen Y., Zhang W., Yu L. (2016). Hydrogen bonding slows down surface diffusion of molecular glasses. J. Phys. Chem. B.

